# Outlier-Based Identification of Copy Number Variations Using Targeted Resequencing in a Small Cohort of Patients with Tetralogy of Fallot

**DOI:** 10.1371/journal.pone.0085375

**Published:** 2014-01-06

**Authors:** Vikas Bansal, Cornelia Dorn, Marcel Grunert, Sabine Klaassen, Roland Hetzer, Felix Berger, Silke R. Sperling

**Affiliations:** 1 Department of Cardiovascular Genetics, Experimental and Clinical Research Center, Charité - Universitätsmedizin Berlin and Max Delbrück Center (MDC) for Molecular Medicine, Berlin, Germany; 2 Department of Mathematics and Computer Science, Free University of Berlin, Berlin, Germany; 3 Department of Biology, Chemistry, and Pharmacy, Free University of Berlin, Berlin, Germany; 4 For the National Register for Congenital Heart Defects, Berlin, Germany; 5 Experimental and Clinical Research Center, Charité - Universitätsmedizin Berlin and Max Delbrück Center (MDC) for Molecular Medicine, Berlin, Germany; 6 Department of Pediatric Cardiology, Charité - Universitätsmedizin Berlin, Berlin, Germany; 7 Department of Cardiac Surgery, German Heart Institute Berlin, Berlin, Germany; 8 Department of Pediatric Cardiology, German Heart Institute Berlin, Berlin, Germany; University of Illinois at Chicago, United States of America

## Abstract

Copy number variations (CNVs) are one of the main sources of variability in the human genome. Many CNVs are associated with various diseases including cardiovascular disease. In addition to hybridization-based methods, next-generation sequencing (NGS) technologies are increasingly used for CNV discovery. However, respective computational methods applicable to NGS data are still limited. We developed a novel CNV calling method based on outlier detection applicable to small cohorts, which is of particular interest for the discovery of individual CNVs within families, *de novo* CNVs in trios and/or small cohorts of specific phenotypes like rare diseases. Approximately 7,000 rare diseases are currently known, which collectively affect ∼6% of the population. For our method, we applied the Dixon’s Q test to detect outliers and used a Hidden Markov Model for their assessment. The method can be used for data obtained by exome and targeted resequencing. We evaluated our outlier- based method in comparison to the CNV calling tool CoNIFER using eight HapMap exome samples and subsequently applied both methods to targeted resequencing data of patients with Tetralogy of Fallot (TOF), the most common cyanotic congenital heart disease. In both the HapMap samples and the TOF cases, our method is superior to CoNIFER, such that it identifies more true positive CNVs. Called CNVs in TOF cases were validated by qPCR and HapMap CNVs were confirmed with available array-CGH data. In the TOF patients, we found four copy number gains affecting three genes, of which two are important regulators of heart development (*NOTCH1*, *ISL1*) and one is located in a region associated with cardiac malformations (*PRODH* at 22q11). In summary, we present a novel CNV calling method based on outlier detection, which will be of particular interest for the analysis of *de novo* or individual CNVs in trios or cohorts up to 30 individuals, respectively.

## Introduction

Many genomic studies have revealed a high variability of the human genome, ranging from single nucleotide variations and short insertions or deletions to larger structural variations and aneuploidies. Structural variations include copy number variations (CNVs), which cause gains (duplications) or losses (deletions) of genomic sequence. These copy number changes are usually defined to be longer than ∼500 bases, including large variations with more than 50 kilobases [1], [2]. Recent studies have identified CNVs associated with a number of complex diseases such as Crohn’s disease, intellectual disability and congenital heart disease [3]–[6].

**Figure 1:**
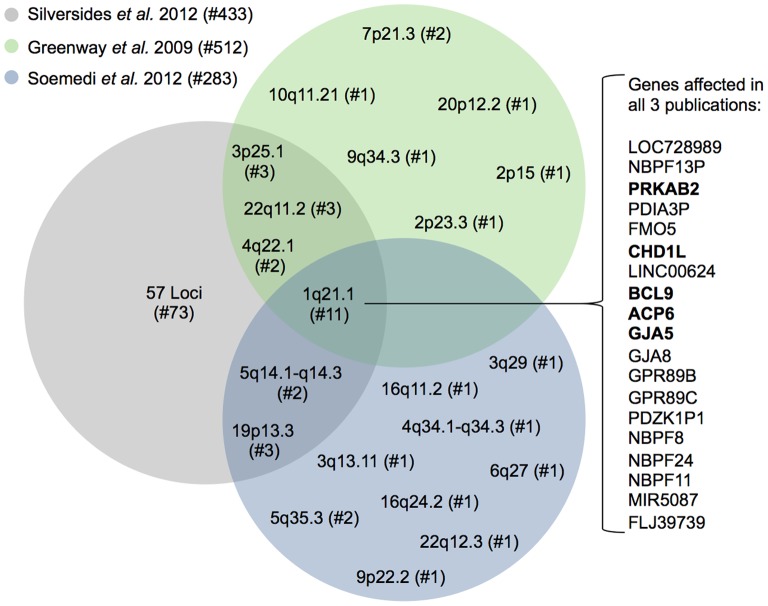
Overlap of three recent CNV studies in TOF patients. All three studies are based on SNP arrays. Loci with detected CNVs are depicted according to their respective cytoband. For 1q21.1, which was identified in all three studies, the RefSeq genes that are affected in at least one patient in each of the publications are listed in the order of their genomic position. Genes that are expressed in mouse heart development (E8.5– E12.0, Mouse Atlas of Gene Expression at http://www.mouseatlas.org/mouseatlas_index_html) are marked in bold. # denotes the number of individuals.

Congenital heart disease (CHD) are the most common birth defect in human with an incidence of around 1% in all live births [7], [8]. They comprise a heterogeneous group of cardiac malformations that arise during heart development. The most common cyanotic form of CHD is Tetralogy of Fallot (TOF), which accounts for up to 10% of all heart malformations [9]. TOF is characterized by a ventricular septal defect with an overriding aorta, a right ventricular outflow tract obstruction and a right ventricular hypertrophy [10]. It is a well-recognized subfeature of syndromic disorders such as DiGeorge syndrome (22q11 deletion), Down syndrome, Holt-Oram syndrome and Williams-Beuren syndrome [11]. Deletions at the 22q11 locus account for up to 16% of TOF cases [12] and copy number changes at other loci were identified in several syndromic TOF patients [13]–[15]. However, the majority of TOFs are isolated, non-syndromic cases caused by a multifactorial inheritance with genetic-environmental interactions, which is also the situation for the majority of CHDs [16]. Using SNP arrays, three recent studies also identified CNVs in large cohorts of non-syndromic TOF patients [17]–[19]. Observing the overlap between these studies with hundreds of cases revealed only one locus (1q21.1) affected in 11 patients (Figure 1), which underlines the heterogeneous genetic background of non-syndromic TOF.

As an alternative to the conventional SNP arrays, next-generation sequencing (NGS) technologies have been widely used to detect single or short sequence variations. The obtained sequence data can also be used to find larger CNVs. Depending on the sequencing technologies, there are different computational approaches for detecting copy numbers from NGS data. For exome sequencing or targeted resequencing, the read-depth or depth of coverage approach is widely used. It assumes that the mapped reads are randomly distributed across the reference genome or targeted regions. Based on this assumption, the read-depth approach analyses differences from the expected read distribution to detect duplications (higher read depth) and deletions (lower read depth) [20]. Applying this approach, several tools have been developed to identify CNVs from exome sequencing data, such as FishingCNV, CONTRA, ExomeCNV, ExomeDepth, XHMM, CoNVEX and CoNIFER [21]–[27].

Here, we aimed to identify copy number alterations in a small cohort of non-syndromic TOF patients based on targeted resequencing data. Assuming a heterogeneous genetic background with individual disease-relevant CNVs, we developed a novel CNV calling method based on outlier detection using Dixon’s Q test and assessment of outliers using a Hidden Markov Model (HMM). For evaluation, we applied our method to a small cohort of HapMap samples and compared it to results obtained with ExomeDepth and CoNIFER. Subsequently, our method and CoNIFER were used to detect CNVs in the TOF patients. Two copy number gains were identified by both methods and are duplications in the *PRODH* gene located at the 22q11 locus. In addition, our outlier-based method found a gain in *NOTCH1* as well as in *ISL1*. All four CNVs could be validated by quantitative real-time PCR.

## Materials and Methods

### Ethics Statement

Studies on TOF patients were performed according to institutional guidelines of the German Heart Institute in Berlin, with approval of the ethics committee of the Charité Medical Faculty and informed written consent of patients and/or parents, kin, caretakers, or guardians on the behalf of the minors/children participants involved in our study.

### TOF Samples and DNA Targeted Resequencing

Targeted resequencing was performed for eight TOF patients, which are unrelated sporadic cases with a well-defined coherent phenotype and no further anomalies. Blood samples (TOF-23, TOF-24, TOF-25, TOF-26, TOF-27) and cardiac tissue from the right ventricle (TOF-01, TOF-02, TOF-18) were collected in collaboration with the German Heart Institute in Berlin and the National Registry of Congenital Heart Disease in Berlin and used for the extraction of genomic DNA. 3–5 µg of genomic DNA were used for Roche NimbleGen sequence capturing using 365 K arrays. For array design, 867 genes and 167 microRNAs (12,910 exonic targets representing 4,616,651 target bases) were selected based on knowledge gained in various projects [28]–[30]. DNA enriched after NimbleGen sequence capturing was sequenced using the Illumina Genome Analyzer (GA) IIx (36 bp paired-end reads). Sequencing was performed by Atlas Biolabs (Berlin) according to manufacturers’ protocols.

**Figure 2:**
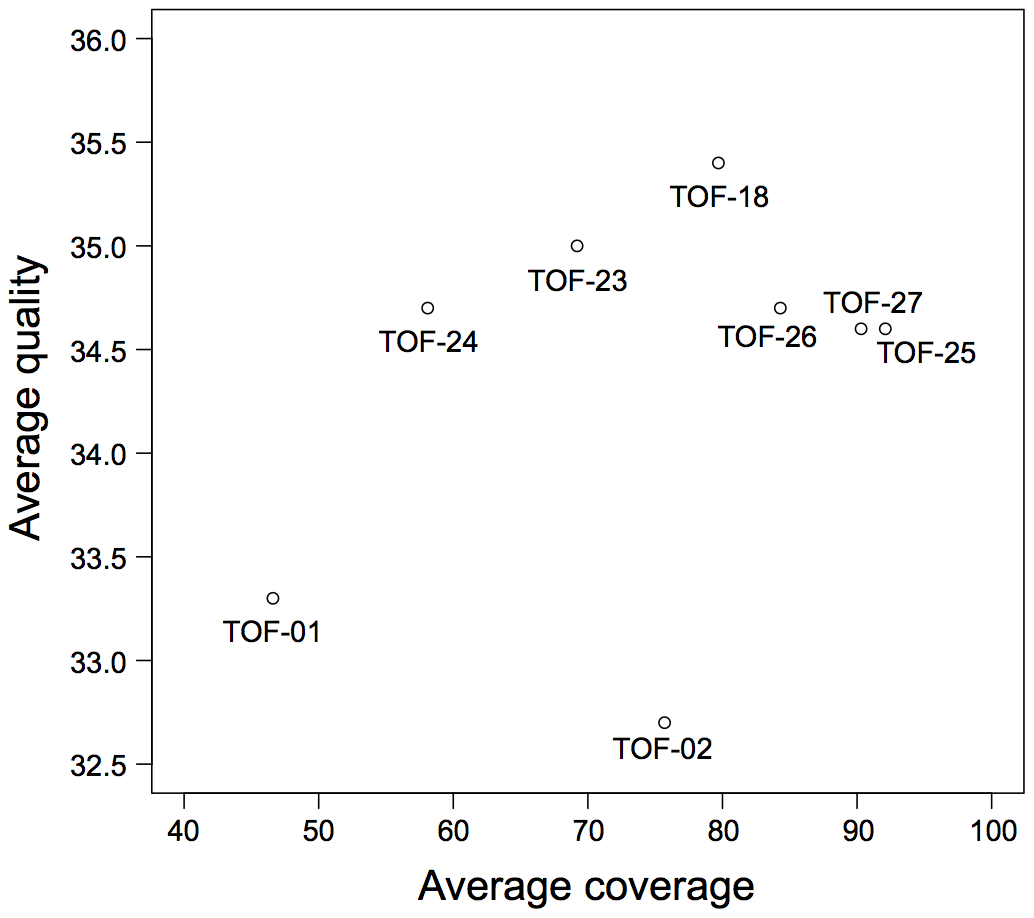
Base qualities versus coverage values. Scatterplot indicates the average base qualities (Phred scores) and depths of coverage for samples targeted resequenced by Illumina’s Genome Analyzer IIx platform (36 bp paired-end reads).

On average, sequencing resulted in 13,331,661 read pairs per sample (Table 1). Average read depths of 75× and base quality scores of 34 (Phred scores) were reached in the captured regions over all samples (Table 1 and Figure 2).

**Table 1:** Number and quality of 36-end reads obtained from targeted resequencing in TOF patients using Illumina’s Genome Analyzer IIx platform.

			Captured regions			
Sample	Number ofreads	Number of readpairs	Phred qualityscore	Mediancoverage	Meancoverage	Target bases with ≥10xcoverage
TOF-01	31,942,782	15,971,391	33.3	40	47	93.85%
TOF-02	26,970,680	13,485,340	32.7	66	76	97.70%
TOF-18	25,476,308	12,738,154	35.4	71	80	98.35%
TOF-23	20,885,192	10,442,596	35.0	60	69	97.41%
TOF-24	25,483,166	12,741,583	34.7	51	58	96.72%
TOF-25	30,551,674	15,275,837	34.6	84	92	98.91%
TOF-26	27,878,750	13,939,375	34.7	75	84	98.34%
TOF-27	24,118,022	12,059,011	34.6	78	90	98.00%

### HapMap Samples

We used exome sequencing data from eight HapMap individuals (NA18507, NA18555, NA18956, NA19240, NA12878, NA15510, NA18517, NA19129). The exomes were captured using Roche NimbleGen EZ Exome SeqCap Version 1 and sequencing was performed using an Illumina HiSeq 2000 platform with 50 bp paired-end reads. The exome sequence data are available from the Short Read Archive at the NCBI (SRA039053). The reads were further trimmed to 36 bp.

### Outlier-based CNV Calling Method

Our CNV calling method was developed for exome or targeted resequencing data of small sets of samples (at least 3 and at most 30) assuming that the bias in the captured regions is similar in all samples enriched and sequenced with the same technology. Based on a heterogeneous genetic background in the cohort, it was further assumed that a unique disease-related copy number change is only present in very few samples.

**Figure 3:**
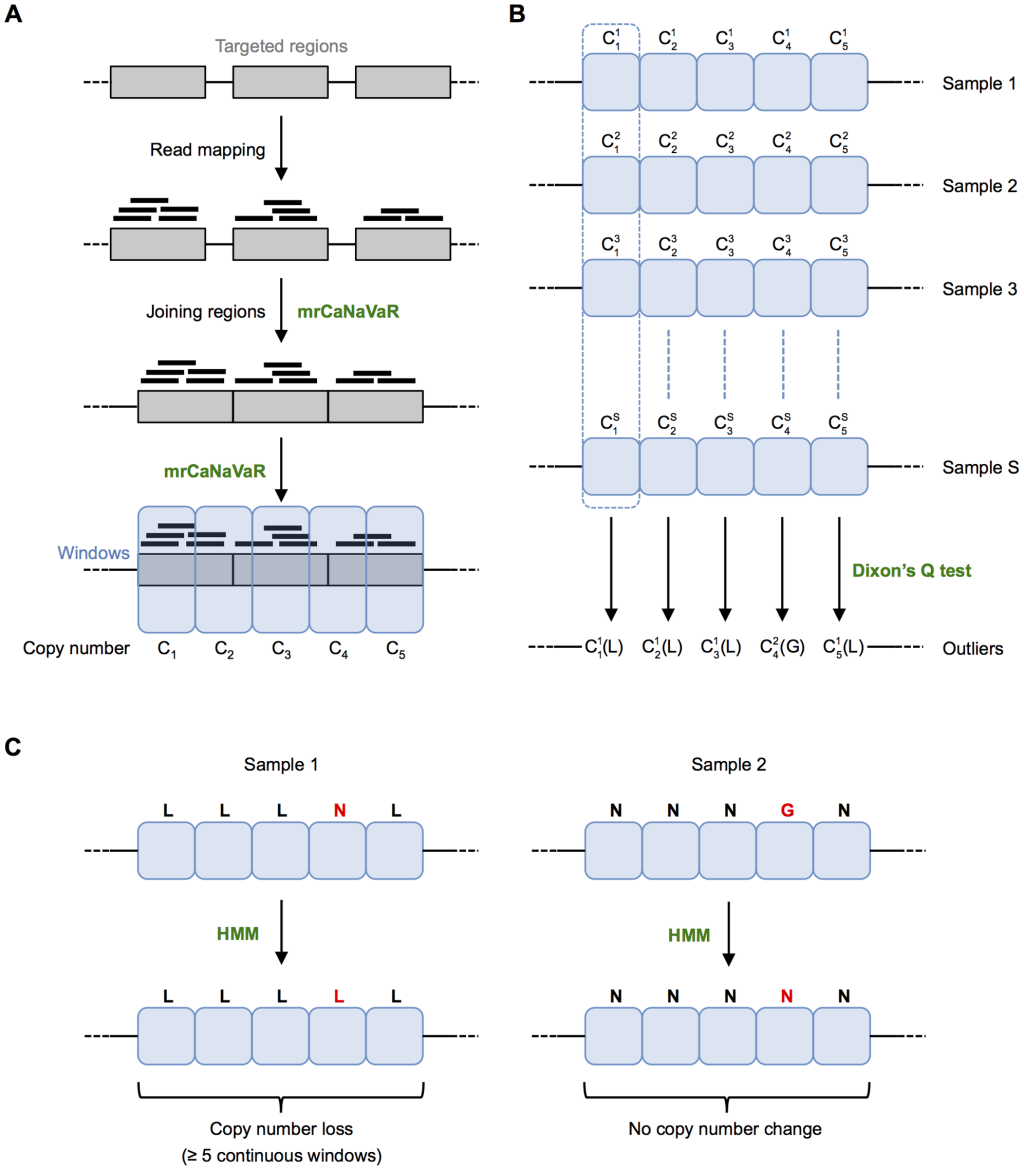
Outlier-based CNV calling method. (A) Read mapping and calculation of copy number value per window. Reads are mapped to extended targeted regions, which are then joined chromosome-wise. mrCaNaVaR is used to split the joined regions into windows. For each window, its copy number value is calculated by mrCaNaVaR, where 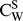 represents the value for window W in sample S. (B) Dixon’s Q test is applied for each window over all samples to identify outliers. Here, sample 1 represents an outlier (loss, L) for the first, second, third and fifth window, while sample 2 represents an outlier (gain, G) for the fourth window. (C) Assessment of outliers using a Hidden Markov Model (HMM). In the given example, the fourth window of sample 1 is considered as normal (N). After applying the HMM, it will also be considered as a loss. Similarly, the fourth window of sample 2 is considered as normal after applying the HMM. A region is called as a copy number alteration, if at least five continuous windows show the same kind of change, i.e. either gain or loss.

First, read mapping and calculation of copy number values were performed for each sample separately. The sequenced reads were mapped to the targeted regions of the reference genome using BWA v.0.5.9 in paired-end mode (‘sampe’) with default parameters [31]. Up- and downstream, the targeted regions (usually exons) were extended by 35 bp (read length minus one base pair) to correctly capture the coverage at the start and end of a region. After mapping, the extended regions with their mapped reads were joined chromosome-wise and the tool mRCaNaVaR v0.34 [32] was used to split the joined regions into non-overlapping windows of 100 bp in length. The copy number value *C* for each window *W*∈{1,…,*n*} of a sample *S*∈{1,…,*n*} was then calculated by mRCaNaVaR using the following formula:
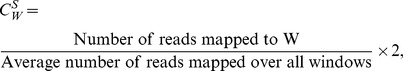
with additional GC correction [32] (Figure 3A). Reads spanning the border of two windows were assigned to the left window. In general, our method calculates a copy number value using mrCaNaVaR, which can accurately predict CNVs with at least 4x coverage [32].

Second, Dixon’s Q test was applied for each window at the same position over all samples to identify gains or losses considered as outliers (Figure 3B). This test was introduced in 1950 for the analysis of extreme values and for the rejection of outlying values [33]. We used the formulas for r_10_ and r_20_ [34], also known as type10 and type20 in the R package ‘outliers’ v0.14 (http://www.R-project.org). Type10 (recommended for 3–7 samples) can only detect a single outlying window at the same genomic position over all samples, while type20 (recommended for 8–30 samples) can identify exactly two outlying windows, meaning the Q test will not detect outliers if more than 2 outliers are present. For each window, we first applied type20, however, if no two significant outliers (samples) were found, type10 was used to detect at most one outlier. Note that our method can also be applied using type10 and type20 independently. Outliers were regarded as significant with a p-value of less than or equal to 0.01. In general, the higher the p-value cutoff, the higher the number of detected outliers but also the number of false positives, i.e. the p-value is a tuning parameter for sensitivity of our method.

In the third and final step, the samples were again considered separately. For each sample, a Hidden Markov Model [35] was applied to get the most likely state of each window (i.e. gain, loss or normal). The initial transition and emission probabilities of the HMM are given in Table S1 and the values were recomputed using the Baum-Welch algorithm [36] implemented in the R package ‘HMM’ v1.0. The most likely sequence of the hidden states was then found by the Viterbi algorithm [37] also implemented in the R package ‘HMM’. Finally, a region was called as copy number gain or loss if at least five continuous windows were considered as a gain or loss, respectively (Figure 3C). This results in a minimum size of 500 bp for detectable CNVs.

We have included a script, written in R 2.15.1 (http://www.R-project.org), for our CNV calling method based on outlier detection in exome and/or targeted resequencing data (Script S1).

### CNV Validation

Genomic DNA was extracted from whole blood or cardiac biopsies using standard procedures. Quantitative real-time PCR was carried out using GoTag qPCR Master Mix (Promega) on an ABI PRISM 7900HT Sequence Detection System (Applied Biosystems) according to the manufacturer’s instructions and with normalization to the *RPPH1* gene. Primer sequences are available on request. As a reference, genomic DNA from the HapMap individual NA10851 was obtained from the Coriell Cell Repositories (New Jersey, USA).

## Results and Discussion

We applied our outlier-based CNV calling method to eight HapMap control samples and intersected our exome-based calls from five of the samples with previously generated calls from high-resolution microarray-based comparative genomic hybridization (array-CGH) [2]. In addition to our method, we used the two publicly available tools ExomeDepth and CoNIFER [23], [27]. Other tools such as CONTRA, FishingCNV, CoNVEX and ExomeCNV could not be applied to this dataset since they need either matched or non-matched controls.

CoNIFER (copy number inference from exome reads) is a method that combines the read-depth approach with singular value decomposition (SVD) normalization to identify rare and common copy number alterations from exome sequencing data [27]. Applying our method with type10 Dixon’s Q test (assuming at most one outlier), we found 40 CNVs over the five HapMap controls (Table S2), out of which 37 regions were also identified in the array-CGH data, showing a high positive predictive value of 93%. With type20 (assuming at most two outliers), we found 65 copy number changes (Table S3), out of which 55 regions are present in the array-CGH data, resulting in a positive predictive value of 85%. Using CoNIFER, 32 CNVs were identified in the five HapMap exome controls and only 26 of these regions are also present in the array-CGH data [27], which corresponds to a positive predictive value of 81% (Table 2). Comparing our results to those obtained from CoNIFER, we found that with type10 16 out of 40 regions (40%) are overlapping with regions called by CoNIFER by at least one base pair. Vice versa, 11 out of 32 regions (34%) overlap with our calls. With type20, 24 out of our 65 called regions (37%) overlap with those from CoNIFER and oppositely, 47% of the regions (15 out of 32) overlap with our calls. In general, CNV regions identified by CoNIFER are longer than those found by our method, meaning that regions called by CoNIFER can correspond to more than one of our CNVs, which explains the different overlap proportions.

**Table 2:** Exome sequencing-based CNV calls in HapMap samples.

Method	Numberof CNVs	Validation dataset	Number ofoverlapping CNVs	Positive predictivevalue	Sensitivity
Outlier-based calling method with type10	40	3,330 arrayCGH calls	37	93%	1.1%
Outlier-based calling method withtype20 including type10	65		55	85%	1.7%
CoNIFER	32		26	81%	0.8%
ExomeDepth	1,555		253	16%	7.6%

Overall, our method was able to detect more copy number changes and has a higher proportion of true positives compared to CoNIFER. However, there is still a large number of CNVs observed in the array-CGH data, which were identified by neither of the two exome-based methods (Table 2). This can for example be explained by their location in segmental duplications and polymorphic but not duplicated regions [27].

ExomeDepth uses a beta-binomial model for the read count data to identify CNVs from exome sequencing data [24]. We applied ExomeDepth with default parameters to the eight HapMap samples and intersected the found CNVs from five of the samples with previously generated calls from array-CGH. In summary, ExomeDepth found 1,555 CNVs in the five samples (median number of 286 CNVs per sample). Out of these, only 253 CNVs overlapped with 3,330 array-CGH calls, which suggest a positive predictive value of 16% and sensitivity of 7.6% (Table 2).

Interestingly, all the five rare CNVs in the five HapMap samples (see Krumm *et al.* 2012, Table S2 [27]) were found by our method, CoNIFER and ExomeDepth. Moreover, ExomeDepth identified more CNVs as compared to CoNIFER and to our method (Table 2), however; the positive predictive value is very low. Therefore, we decided not to use ExomeDepth for detecting CNVs in the TOF patients.

**Figure 4:**
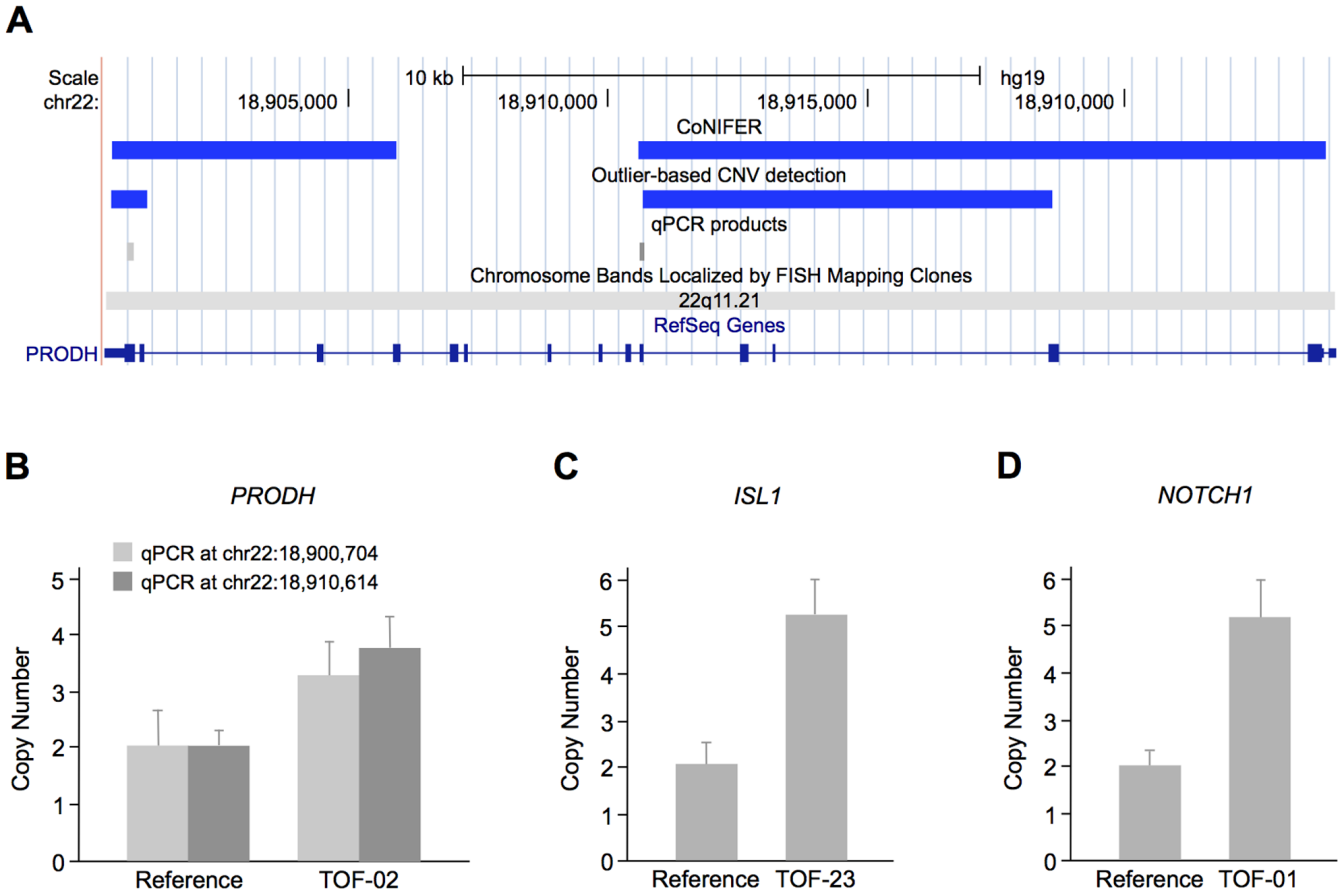
CNVs in TOF patients. (A) CNVs detected in *PRODH* by CoNIFER and our outlier-based CNV calling method. The duplications are depicted in the UCSC Genome Browser as blue bars. The positions of the two quantitative real-time PCR products selected for validation are shown as light and dark grey bars, respectively. (B) Quantitative real-time PCR validation of *PRODH* copy number gains. Measurement was performed at two different positions (light and dark grey bars, respectively) and normalized to the *RPPH1* gene. The HapMap individual NA10851 was used as a reference. The plot shows a representative of two independent measurements, which were each performed in triplicates. (C–D) Validation of copy number gains in *ISL1* and *NOTCH1*, respectively, that were only identified by our outlier-based CNV calling method.

To identify copy number alterations in TOF patients, we applied our outlier-based method as well as CoNIFER to targeted resequencing data of our eight cases. Using our method, we found four copy number gains in three genes, namely *ISL1*, *NOTCH1* and *PRODH*. CoNIFER only identified two gains in *PRODH*, which overlap with the two regions found by our method (Table 3 and Figure 4A). We further validated all four regions identified by our method using quantitative real-time PCR (Figure 4B–D). ISL1 is a homeobox transcription factor that marks cardiovascular progenitors [38] and is known to be associated with human congenital heart disease [39]. NOTCH1 is a transmembrane receptor involved in the NOTCH signaling pathway, which plays a crucial role in heart development [40]. Mutations in *NOTCH1* are associated with a spectrum of congenital aortic valve anomalies [41], [42] and a copy number loss was identified in a patient with TOF [17] (locus 9q34.3, Figure 1). The mitochondrial protein PRODH catalyzes the first step in proline degradation and is located in the 22q11.2 locus. Deletions in this region are associated with the DiGeorge syndrome and 80% of cases harbor cardiovascular anomalies [43]. A copy number gain and two losses in the 22q11.2 locus overlapping *PRODH* were also identified in sporadic TOF patients [17], [18] (Figure 1).

**Table 3:** Targeted resequencing-based CNV calls in TOF patients.

Method	Type of variation	Position (hg19)	Length in bp	Gene	Sample
Outlier-based calling method withtype20 including type10	Gain	chr5∶50,689,340–50,689,940	601	*ISL1*	TOF-23
	Gain	chr9∶139,402,477–139,404,228	1,752	*NOTCH1*	TOF-01
	Gain	chr22∶18,900,412–18,901,127	716	*PRODH*	TOF-02
	Gain	chr22∶18,910,691–18,918,575	7,885	*PRODH*	TOF-02
CoNIFER	Gain	chr22∶18,900,414–18,905,939	5,526	*PRODH*	TOF-02
	Gain	chr22∶18,910,575–18,923,866	13,292	*PRODH*	TOF-02

In summary, we developed an outlier-based CNV calling method for a small cohort size of up to 30 individuals. The exploration of the human phenotype and its genetic and molecular background is the challenge of the next century and it is already clear that more precise phenotyping will lead to smaller cohort sizes. Here, novel approaches will be of exceptional relevance. Moreover, analyzing small patient cohorts is of special interest for rare diseases with only few available patient samples. Approximately 7,000 rare diseases are currently known and together affect about 6% of the population [44]. Our method is based on the assumption that individual CNVs (outliers) are disease-relevant and can be applied to exome as well as targeted resequencing data. Both sequencing techniques achieve a high read coverage over the targeted regions. Nevertheless, there are non-uniform patterns in the read depth resulting mainly from repetitive regions. Thus, the detection of copy number alterations is limited in these genomic regions, which is shown by the high number of false negatives compared to array-CGH [27].

We evaluated our method using publicly available data of eight HapMap samples and subsequently applied it to a small number of TOF patients. Compared to CoNIFER we identified more CNVs in both the HapMap samples as well as in our TOF cohort. In general, our method assumes a uniform read distribution over all exons of all individuals enriched and sequenced with the same technology to compare read counts between all samples to detect outliers. In contrast, CoNIFER considers the read depth across all individuals after SVD normalization. This difference is also reflected by the overlap of their calls in the eight HapMap samples. Although the general overlap is relatively low, we were able to identify all rare CNVs detected by CoNIFER. In addition to searching for rare CNVs, we also found a subset of common CNVs called by CoNIFER. This might be explained by variations present in only one or two of the eight individuals, but defined as common based on their frequency in a larger population.

In our TOF cohort comprising eight cases, we found four copy number gains in three patients, while CoNIFER only detected two of the gains in one patient. All four gains could be validated and in addition, the three genes affected by the CNVs are important regulators of heart development (*NOTCH1*, *ISL1*) or are located in a region associated with cardiac malformations (*PRODH*). Two of the variations also overlap with copy number alterations in TOF patients previously identified by array-CGH [17], [18]. Taken together, this illustrates the advantage of using an outlier-based detecting method in a small cohort with a heterogeneous genetic background. Thus, our method is of special interest for small cohorts of specific phenotypes like rare diseases. Moreover, it can be used for the discovery of individual CNVs within families and *de novo* CNVs in trios.

## Supporting Information

Table S1 **Initial transition and emission probabilities of the HMM.**
(PDF)

Table S2 **CNVs found in the five HapMap samples using type10 Dixon’s Q test in the outlier-based CNV calling method.**
(PDF)

Table S3 **CNVs found in the five HapMap samples using type20 Dixon’s Q test in the outlier-based CNV calling method.**
(PDF)

Script S1 **R script for CNV calling.**
(TXT)
