# Supplementary material for: Outlier-Based Identification of Copy Number Variations Using Targeted Resequencing in a Small Cohort of Patients with Tetralogy of Fallot
Source: PLoS One. 2014 Jan 6;9(1):e85375. doi: 10.1371/journal.pone.0085375 (PMC3882271; doi:10.1371/journal.pone.0085375)
Supplement: Table S1 — Initial transition and emission probabilities of the HMM. (PDF) [file pone.0085375.s001.pdf]

Table S1. Initial transition and emission probabilities of the HMM.

	Gain	Loss	Normal
**Gain**	0.6	0.2	0.2
**Loss**	0.2	0.6	0.2
**Normal**	0.2	0.2	0.6
