# Supplementary material for: Outlier-Based Identification of Copy Number Variations Using Targeted Resequencing in a Small Cohort of Patients with Tetralogy of Fallot
Source: PLoS One. 2014 Jan 6;9(1):e85375. doi: 10.1371/journal.pone.0085375 (PMC3882271; doi:10.1371/journal.pone.0085375)
Supplement: Table S2 — CNVs found in the five HapMap samples using type10 Dixon’s Q test in the outlier-based CNV calling method. (PDF) [file pone.0085375.s002.pdf]

Table S2. CNVs found in the five HapMap samples using type10 Dixon's Q test in the outlier-based CNV calling method.

Chr	Start position (hg19)	End position (hg19)	Type of variation	HapMap sample
chr1	155,234,407	155,237,870	gain	NA15510
chr1	155,253,768	155,261,736	gain	NA15510
chr2	240,981,511	240,982,011	gain	NA12878
chr3	19,559,462	19,924,248	gain	NA15510
chr3	20,164,156	20,181,845	gain	NA15510
chr3	20,215,780	20,216,280	gain	NA15510
chr4	68,795,606	68,925,183	gain	NA18517
chr4	68,928,187	68,928,787	gain	NA18517
chr4	68,930,393	68,934,496	gain	NA18517
chr5	69,717,189	69,718,089	gain	NA18517
chr5	69,729,631	69,730,131	gain	NA18517
chr5	70,308,153	70,308,653	gain	NA18517
chr7	99,564,684	99,621,311	gain	NA15510
chr9	108,456,919	108,536,213	gain	NA15510
chr9	117,087,073	117,092,300	gain	NA15510
chr9	40,773,663	40,774,263	gain	NA12878
chr9	41,590,682	41,592,182	gain	NA12878
chr11	4,967,401	4,968,201	gain	NA19240
chr11	5,878,066	5,878,966	loss	NA19240
chr11	6,190,624	6,191,524	loss	NA19129
chr12	133,721,045	133,733,489	gain	NA19240
chr12	133,764,519	133,768,587	gain	NA19240
chr12	133,778,781	133,779,381	gain	NA19240
chr14	106,539,004	106,539,504	gain	NA19240
chr14	106,780,499	106,781,099	gain	NA19240
chr14	21,359,867	21,423,999	loss	NA19240
chr16	21,623,981	21,636,326	gain	NA18517
chr16	21,658,494	21,666,721	gain	NA18517
chr16	21,702,877	21,712,336	gain	NA18517
chr16	21,734,219	21,739,705	gain	NA18517
chr17	39,535,858	39,538,575	gain	NA19240
chr17	44,171,932	44,249,515	gain	NA12878
chr19	43,688,932	43,698,720	gain	NA18517
chr19	9,868,776	9,869,276	loss	NA19129
chr22	20,457,890	20,459,090	gain	NA19129
chr22	21,742,009	21,742,909	gain	NA19129
chr22	21,828,820	21,829,620	gain	NA19129
chr22	21,900,797	21,901,297	gain	NA19129
chr22	22,453,213	22,453,713	loss	NA12878
chr22	23,134,983	23,135,483	loss	NA12878
