# Supplementary material for: Outlier-Based Identification of Copy Number Variations Using Targeted Resequencing in a Small Cohort of Patients with Tetralogy of Fallot
Source: PLoS One. 2014 Jan 6;9(1):e85375. doi: 10.1371/journal.pone.0085375 (PMC3882271; doi:10.1371/journal.pone.0085375)
Supplement: Table S3 — CNVs found in the five HapMap samples using type20 Dixon’s Q test in the outlier-based CNV calling method. (PDF) [file pone.0085375.s003.pdf]

**Table S3. CNVs found in the five HapMap samples using type20 Dixon's Q test in the outlier-based CNV calling method.**

| Chr | Start position (hg19) | End position (hg19) | Type of variation | HapMap sample |
|---|---|---|---|---|
| chr1 | 152,573,211 | 152,586,435 | loss | NA15510 |
| chr1 | 152,573,211 | 152,586,435 | loss | NA19129 |
| chr1 | 155,234,407 | 155,237,870 | gain | NA15510 |
| chr1 | 155,253,768 | 155,261,736 | gain | NA15510 |
| chr2 | 240,981,511 | 240,982,311 | gain | NA12878 |
| chr3 | 19,559,462 | 19,930,107 | gain | NA15510 |
| chr3 | 20,164,156 | 20,187,926 | gain | NA15510 |
| chr3 | 20,215,780 | 20,216,280 | gain | NA15510 |
| chr4 | 68,795,606 | 68,925,183 | gain | NA18517 |
| chr4 | 68,928,187 | 68,928,787 | gain | NA18517 |
| chr4 | 68,930,393 | 68,934,496 | gain | NA18517 |
| chr4 | 70,146,232 | 70,146,832 | loss | NA12878 |
| chr4 | 70,146,232 | 70,146,932 | loss | NA19129 |
| chr4 | 70,152,473 | 70,160,559 | loss | NA12878 |
| chr4 | 70,152,473 | 70,160,559 | loss | NA19129 |
| chr5 | 69,717,189 | 69,718,089 | gain | NA18517 |
| chr5 | 69,729,631 | 69,730,131 | gain | NA18517 |
| chr5 | 69,733,151 | 69,733,651 | gain | NA18517 |
| chr5 | 70,308,153 | 70,308,753 | gain | NA18517 |
| chr7 | 141,755,347 | 141,758,103 | loss | NA12878 |
| chr7 | 75,045,612 | 75,046,112 | gain | NA19129 |
| chr7 | 99,564,684 | 99,621,311 | gain | NA15510 |
| chr9 | 108,456,919 | 108,536,213 | gain | NA15510 |
| chr9 | 117,087,073 | 117,092,300 | gain | NA15510 |
| chr9 | 40,773,663 | 40,774,263 | gain | NA12878 |
| chr9 | 41,590,682 | 41,592,182 | gain | NA12878 |
| chr11 | 4,967,401 | 4,968,301 | gain | NA19240 |
| chr11 | 5,878,066 | 5,878,966 | loss | NA19240 |
| chr11 | 6,190,624 | 6,191,524 | loss | NA19129 |
| chr11 | 7,817,616 | 7,818,416 | loss | NA19129 |
| chr11 | 7,817,616 | 7,818,416 | loss | NA19240 |
| chr12 | 133,721,045 | 133,733,489 | gain | NA19240 |
| chr12 | 133,764,519 | 133,768,587 | gain | NA19240 |
| chr12 | 133,778,781 | 133,779,381 | gain | NA19240 |
| chr14 | 105,417,358 | 105,418,158 | loss | NA12878 |
| chr14 | 105,417,358 | 105,418,158 | loss | NA19129 |
| chr14 | 106,539,004 | 106,539,504 | gain | NA19240 |
| chr14 | 106,780,499 | 106,781,099 | gain | NA19240 |
| chr14 | 21,359,867 | 21,423,999 | loss | NA19240 |
| chr15 | 22,368,674 | 22,369,374 | gain | NA15510 |
| chr15 | 22,368,674 | 22,369,374 | gain | NA19240 |
| chr15 | 22,466,012 | 22,466,512 | gain | NA15510 |
| chr15 | 22,466,012 | 22,466,512 | gain | NA19240 |

| | | | | |
|---|---|---|---|---|
| chr15 | 22,489,704 | 22,490,204 | gain | NA15510 |
| chr16 | 21,623,981 | 21,636,326 | gain | NA18517 |
| chr16 | 21,658,494 | 21,666,721 | gain | NA18517 |
| chr16 | 21,702,877 | 21,712,336 | gain | NA18517 |
| chr16 | 21,734,219 | 21,739,705 | gain | NA18517 |
| chr16 | 72,107,785 | 72,110,923 | gain | NA18517 |
| chr16 | 72,107,785 | 72,110,923 | gain | NA19240 |
| chr17 | 39,535,858 | 39,538,575 | gain | NA19240 |
| chr17 | 44,171,932 | 44,249,515 | gain | NA12878 |
| chr19 | 43,688,932 | 43,698,720 | gain | NA18517 |
| chr19 | 9,868,176 | 9,869,276 | loss | NA19129 |
| chr22 | 20,456,590 | 20,457,090 | gain | NA19129 |
| chr22 | 20,457,690 | 20,459,090 | gain | NA19129 |
| chr22 | 21,739,909 | 21,740,409 | gain | NA19129 |
| chr22 | 21,742,009 | 21,743,009 | gain | NA19129 |
| chr22 | 21,828,820 | 21,829,620 | gain | NA19129 |
| chr22 | 21,830,142 | 21,831,242 | gain | NA19129 |
| chr22 | 21,832,798 | 21,834,188 | gain | NA19129 |
| chr22 | 21,841,563 | 21,842,863 | gain | NA19129 |
| chr22 | 21,900,797 | 21,901,397 | gain | NA19129 |
| chr22 | 22,453,213 | 22,453,713 | loss | NA12878 |
| chr22 | 23,134,983 | 23,135,483 | loss | NA12878 |
